# EP300 as an oncogene correlates with poor prognosis in esophageal squamous carcinoma

**DOI:** 10.7150/jca.34261

**Published:** 2019-08-29

**Authors:** Yanghui Bi, Pengzhou Kong, Ling Zhang, Heyang Cui, Xiaoqin Xu, Feiyun Chang, Ting Yan, Jiayi Li, Caixia Cheng, Bin Song, Xia Niu, Xiangchen Liu, Xue Liu, Enwei Xu, Xiaoling Hu, Yu Qian, Fang Wang, Hongyi Li, Yanchun Ma, Jian Yang, Yiqian Liu, Yuanfang Zhai, Yi Wang, Yingchun Zhang, Haiyan Liu, Jing Liu, Jintao Wang, Yongping Cui, Xiaolong Cheng

**Affiliations:** 1 Department of Pathology & Shanxi Key Laboratory of Carcinogenesis and Translational Research of Esophageal Cancer, Shanxi Medical University, Taiyuan, Shanxi 030001, P.R. China; 2 Department of Pathology, Shanxi Medical University, Taiyuan, Shanxi 030001, P.R.China; 3 Department of Etiology, Shanxi Cancer Hospital, Taiyuan, Shanxi030001, P.R.China; 4 Department of General Surgery, Shanxi Cancer Hospital, Taiyuan, Shanxi 030001, P.R. China; 5 Anglo-Chinese School (Independent), Singapore; 6 Department of Pathology, The First Hospital, Shanxi Medical University, Taiyuan, Shanxi 030001, P.R. China; 7 Department of Oncology, The First Hospital, Shanxi Medical University, Taiyuan, Shanxi 030001, P.R. China; 8 Department of Pathology, Shanxi Cancer Hospital, Taiyuan, Shanxi 030001, P.R. China; 9 Department of Pharmacology, Shanxi Medical University, Taiyuan, Shanxi 030001, P.R. China; 10 Department of Nuclear Medicine, The First Hospital, Shanxi Medical University, Taiyuan, Shanxi 030001, P.R. China; 11 Department of General Surgery, The First Hospital, Shanxi Medical University, Taiyuan, Shanxi 030001, P.R. China; 12 Department of Epidemiology, School of Public Health, Shanxi Medical University, Taiyuan 030001, P.R. China.; 13 Department of Anatomy, Shanxi Medical University, Taiyuan, Shanxi 030001, P.R. China

**Keywords:** EP300, ESCC, prognosis, angiogenesis, EMT

## Abstract

E1A Binding Protein P300 (EP300) is one of the mutations of genes involved in histone modifications in esophageal squamous cell carcinoma (ESCC). However, its clinical relevance, potential function and mechanisms have remained elusive.

**Methods**: Genomic sequencing datas from 325 esophageal squamous cell carcinoma (ESCC) cases were integrated and screened a series of frequently mutated histone modifier genes. EP300 was selected to further analyze its clinical significance, function and RNA-sequencing was performed to explore its potential mechanism.

**Results**: Of 35 histone modifier genes, EP300 was not only a significantly mutated gene but also a frequently mutated gene with a mutation frequency of more than 10% in ESCC. EP300 mutation was associated with tumor grade, pathological T stage and lymph node metastasis, predicting a shorter cumulative survival status. Immunohistochemical analysis showed that EP300 expression was significantly higher in ESCC tumor tissues, and the expression levels were associated with poor survival of ESCC patients. Moreover, we found that EP300 knockdown led to inhibition of cell proliferation, colony formation, migration and invasion. RNA-sequencing showed EP300 knockdown led to a significant change of genes expression associated with angiogenesis, hypoxia and epithelial-to-mesenchymal transition (EMT).

**Conclusions**: Taken together, our study identified a novel role and mechanism of EP300 in ESCC and provided epigenetic therapeutic strategies for the treatment of ESCC.

## Introduction

Esophageal cancer is the sixth most lethal cancer worldwide with more than 400,000 deaths each year and approximately 70% of global cases occurring in China, where the dominant histologic type is esophageal squamous cell carcinoma (ESCC) 1. Unlike other gastrointestinal tumors such as gastric cancer and colon cancer that have been extensively studied, the achievements of ESCC remains unchanged in the past few decades, with limited clinical approaches for early diagnosis and a five-year survival rate ranging from 15% to 25%^2^. However, most recently, a succession of great achievements has been made in ESCC cancer genomics with the advent and progression of high-throughput next-generation sequencing (NGS) 3-7. The new generation of sequencing technology not only can be no preferences to reflect genomic abnormalities in the overall picture, but also able to detect the high frequency, low frequency and rare alterations closely related to diseases 8.

In our previous study, we performed whole genome sequencing (WGS) of 14 and whole exome sequencing (WES) of 90 pairs of ESCC tumors and matched normal tissue, which revealed the landscapes of driver genes and disrupted pathways in ESCC cases collected from patients of the Taihang Mountains of north-central China 3. Meanwhile, three other large-scale WGS/WES studies of ESCC cohorts have been carried out in China and a WES study in Japan 4-7. These studies not only validated mutations of known cancer-related genes including TP53, CDKN2A, FAT1, NOTCH1, PIK3CA, EGFR, KMT2D, NFE2L2, but also identified several new recurrent alterations including ZNF750, AJUBA, FAM135B, TET2, XPO1 in ESCC. The combination analysis of these genomics sequencing data undoubtedly elucidates the molecular basis underlying the ESCC and guide the development of effective targeted therapies.

In this study, we presented the landscape of genomic alterations in histone modifier genes, which had been recurrently mutated in ESCC identified by combination analysis of NGS studies of four Chinese ESCC cohorts. Furthermore, we explored the correlation between mutation and expression of EP300 and tumor progression and metastasis, as well as its prognostic value for patients with ESCC. Moreover, the function and mechanism of EP300 in ESCC were also reported.

## Results

### The landscape of somatic mutations of histone modifier genes in ESCC

**Figure 1:**
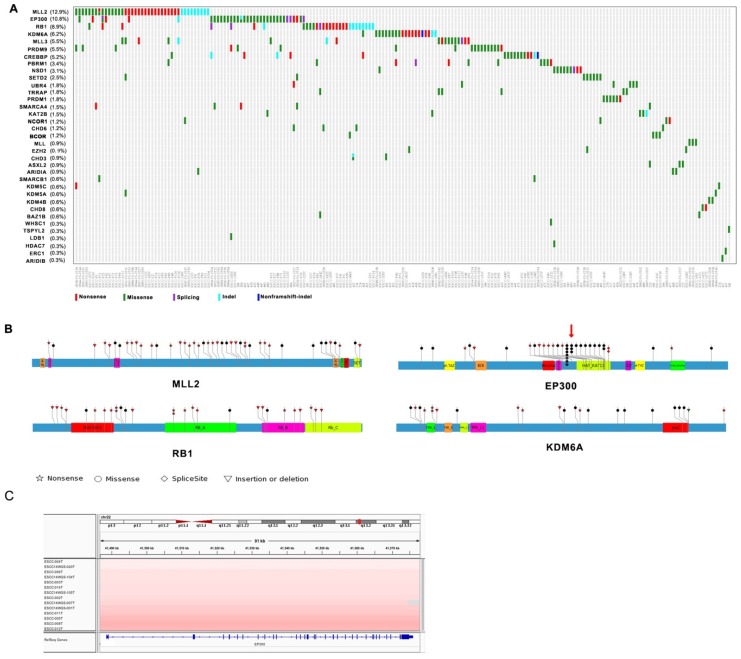
Somatic Mutations of Histone Modifier Genes in ESCC identified by genomic sequencing. **(A)** The panel shows the matrix of mutations in mutated histone modifier genes colored by the mutation subtypes. Each column denotes an individual tumor, and each row represents a gene. The mutated histone modifier genes are shown on the left and the corresponding mutation frequency are marked in brackets. **(B)** A schematic representation of the domain structure of MLL2, EP300, RB1, and shows the location of somatic variants identified in ESCC tumors. **(C)** Focal amplifications at the EP300 locus (22q13.2) in ESCC tumors are shown from IGV.

Among genes mutated in four Chinese cohort cases and previously associated with cancer, thirty-five genes including MLL2, EP300, RB1, KDM6A, MLL3, PRDM9, CREBBP, PBRM1, NSD1, SETD2 are epigenetic modifiers. A range of mutations involving these genes affected 61% of the 325 next generation- sequenced ESCC cases (**Figure 1A**). Interestingly, most of mutations identified in chromatin-remodeling regulators are uncommon in esophageal adenocarcinoma (EAC) but are enriched in ESCC. Of these histone modifier genes, seven genes including MLL2 (12.9%), EP300 (10.8%), RB1 (8.9%), KDM6A (6.2%), MLL3 (5.5%), PRDM9 (5.5%), CREBBP (5.2%) have higher mutation frequency more than 5% in ESCC (**Figure 1A**). EP300, the transcriptional co-activator and histone acetyltransferase, was identified as both frequently mutated gene and significantly mutated gene 3, 4. Of the 37 driver mutations, there are 5 nonsense, 26 missense, 2 frameshift indels, 4 splicing site mutation, and mutations are in general clustered in exons encoding its histone acetyltransferase (HAT) domain. Moreover, nearly 50% of the missense mutations in EP300 are at the three identical amino acid, c.G4195: p.D1399 (7/26, 26.9%) followed by c.4241A: p.Y1414 (3/26, 8.6%) and c.4540G: p.E1514 (2/35, 5.7%) (**Figure 1B**). It is known that the coordination of gene transcription is controlled by a variety of histone modifications including acetylation, phosphorylation, methylation, and ubiquitination. EP300 that regulates transcription via chromatin remodeling is capable of acetylating all the four histones and mediates cAMP-gene regulation by binding specifically to phosphorylated CREBBP protein 8. And we also identified CREBBP to be mutated in 5.5% ESCC cases. Most intriguingly, we observed a significant phosphorylated site at Y1414C on EP300 (*p* < 0.0001, FDR < 0.047), for that was also identified as a harmful mutation. Y1414C located in KAT11 domain which is required for H3K56 acetylation (**Figure 1B**), suggesting that phosphorylated modification may be a possible mechanism for EP300 dysregulation. In addition, EP300 was also found to be amplified in 13 cases out of 31WGS dataset (41.9%, G score < 0.05) (**Figure 1C**). Collectively, the variation of EP300 may be involved in the tumorigenesis and development of esophageal cancer.

The other most common target of alterations was MLL2 with 12.9% cases harboring mutations. Its homolog MLL3 was found to be mutated in 5.5% out of 325 cases. MLL2 and MLL3 belong to the SET1/MLL family, encode histone lysine methyltransferases that play important roles in epigenetic regulation of gene transcription. Interestingly, in ESCC, the mutations of MLL2 were clearly inactivating events represented by nonsense mutations (n = 18, 42.9%), frameshift insertions/deletions (n = 9, 21.4%). MLL3 also involved five nonsense and ten missense mutations in eighteen samples with mutation frequency of 5.5% (**Figure 1A** and** 1B**). In particular, the corresponding mutations are predicted to generate truncated proteins lacking the entire C-terminal cluster of conserved domains (including the SET domain) or significant portions of protein, thus disrupt their function and consequently deregulate the control of chromatin-based processes, ultimately leading to oncogenic transformation and the development of cancer. Recent cancer genetics studies have uncovered frequent somatic loss-of-function mutations in the genes encoding MLL2/3 complex subunits in a variety of cancer types 9, 10. KDM6A, a member of demethyltransferases interacts with MLL2, was also identified to be mutated in 6.2% of ESCC.

### EP300 mutation predicted a shorter survival rate

**Figure 2:**
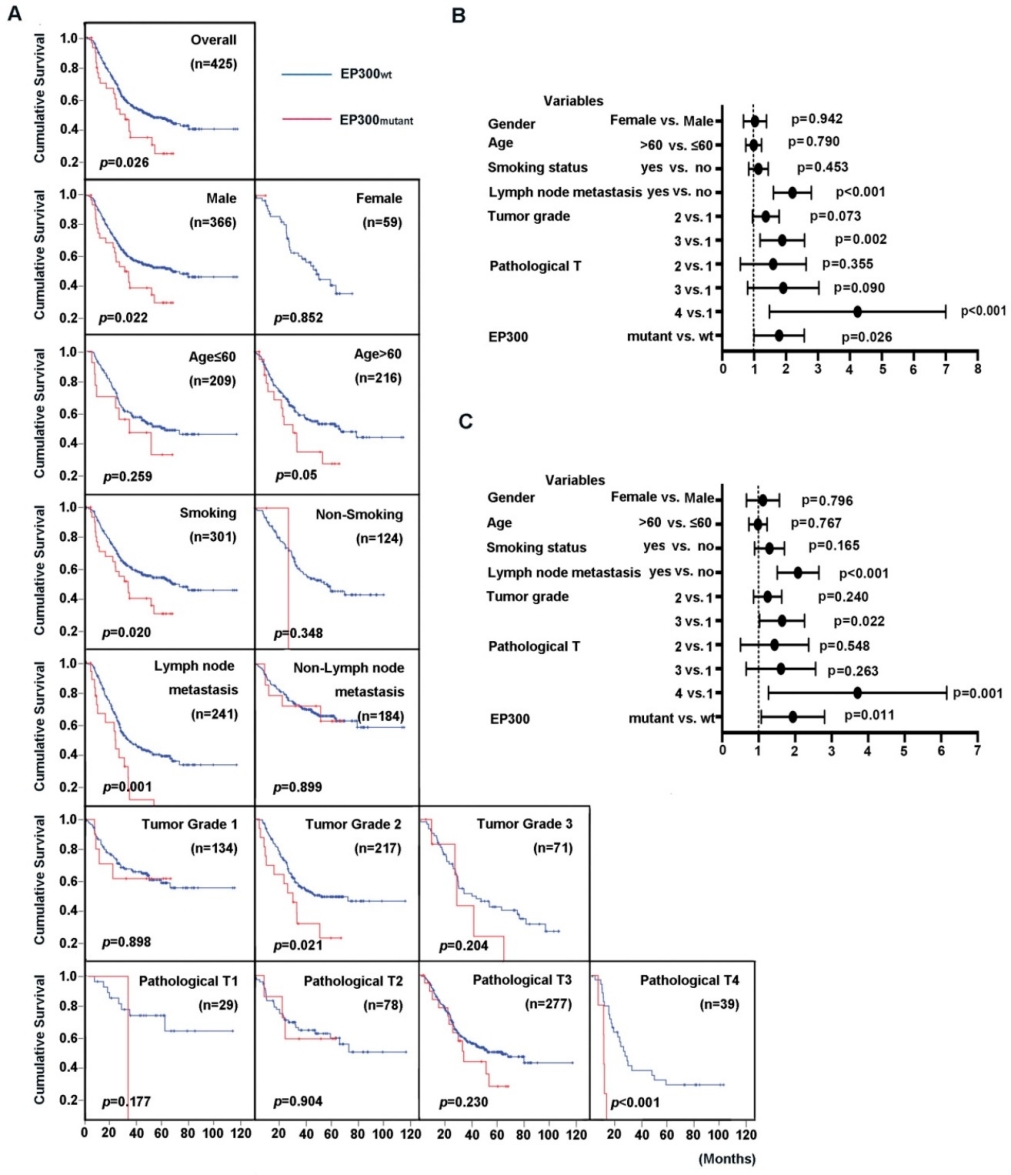
Prognostic value of ESCC patients with different EP300 genotypes. **(A)** Kaplan-Meier survival curves of patients with different EP300 genotypes in overall population and in patients with different age, gender, smoking history, lymph node metastasis, tumor grade and pathological T.** (B)** Univariate analysis by Cox proportional hazards regression model in overall population. **(C)** Multivariate analysis by Cox proportional hazards regression model in overall population.

EP300 was identified to be not only a significantly mutated gene but also a highly frequently mutated gene with mutation frequency more than 10% in ESCC 3, 4. We then integrated four genomic sequencing data and used Cox regression analysis to assess the impact of EP300 mutations and clinical parameters on overall survival (OS). The results showed that the patients with EP300 mutation have poor survival time than those with wild type EP300 (EP300WT) (*p* = 0.026, **Figure 2A**). The association of clinical parameter with OS were statistically significant in regard to tumor grade, pathological T stage and lymph node metastasis. To be specific, the patients of pathological grade 2 with mutant EP300 (EP300mutant) had a shorter OS time than those with wild type EP300 (*p* = 0.021, **Figure 2A**). In patients with lymph node metastasis and pathological T4, EP300mutant group had a shorter OS time than EP300WT group (*p* = 0.001 and *p* < 0.001, **Figure 2A**). Moreover, either for male patients or smokers with mutated EP300 had significantly shorter cumulative survival time (*p* < 0.05, **Figure 2A**).

To evaluate the predictive value of EP300 mutation for cumulative survival status in ESCC patients, we performed univariate and multivariate analysis by Cox proportional hazard regression model. The univariate analysis showed that EP300 mutant, tumor grade, lymph node metastasis and pathological T were significant risk factors for OS status (HR, 1.668; 95% CI, 1.062-2.621; *p* = 0.026) (**Figure 2B, Supplementary Table 1**). The further multivariate analysis drew the same conclusion that EP300 mutation was a significant risk factor for predicting cumulative survival status in the overall study data (HR, 1.809; 95% CI, 1.143-2.864; *p* = 0.011) (**Figure 2C, Supplementary Table 1**).

### High EP300 expression level reflected shorter cumulative survival status in ESCC patients

**Figure 3:**
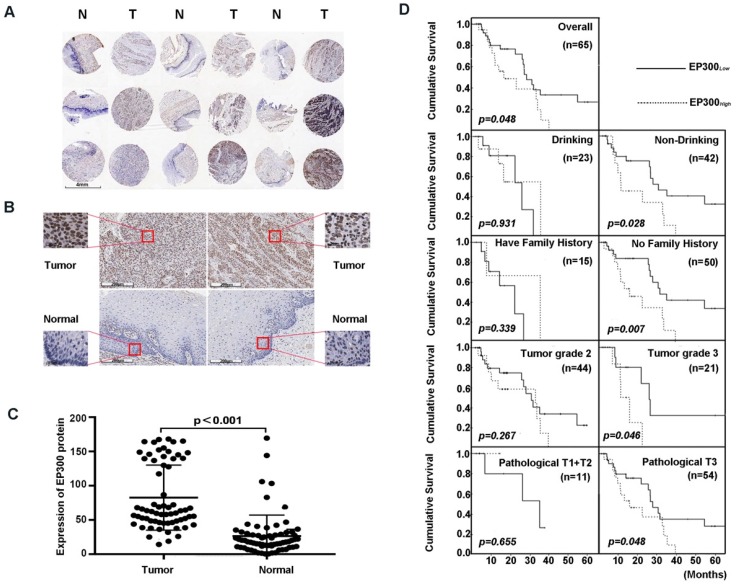
EP300 was frequently up-regulated in ESCC tissues compared to that of adjacent normal tissues. **(A)** and **(B)** Representative immunohistochemistry images of EP300 expression in tumor tissues and adjacent normal tissues from paraffin-embedded formalin-fixed ESCC tissue microarrays containing 65 tumors and corresponding non-tumor tissues. Scale bars represent 200μm. **(C)** Comparison of EP300 protein level in paired ESCC tumor tissues and normal tissues based on TMA data (n = 65, Rank sum test, p < 0.001). **(D)** Kaplan-Meier survival curves of patients with different EP300 level in overall population and in patients with different drinking history, family history, tumor grade and pathological T.

Next, we analyzed the expression level of EP300 by immunohistochemical analysis based on tissue microarray including 65 of ESCC tumor tissues and paired normal tissues (**Figure 3A**). Our results demonstrated that EP300 protein showed strong nuclear staining in esophageal carcinoma tissues whereas nearly negative in matched normal tissues (**Figure 3B**). A significant statistical difference was found between the two groups (*p* < 0.001) (**Figure 3C**). Moreover, according to the receiver operating characteristic curve (ROC) analysis, the optimal cut-off value of 122 of EP300 protein level was selected with higher sensitivity and specificity to divide all ESCC cases into two groups: EP300_low_ (≤ 122) and EP300_high_ (> 122). Our analysis showed that EP300 expression level was associated with gender (*p* = 0.016) and smoking (*p* = 0.022) (**Table 1**). No significant correlations were seen in patients of different age, alcohol history, histological grade, clinical stage or lymph node metastasis (**Table 1**).

We also investigated the relationship between EP300 protein levels and OS time status in ESCC. The OS time of EP300_low_ patients ranged from 4.2 months to 65.3 months, and the median was 27.8 months. The OS time of EP300_high_ ranged from 2.7 months to 39.5 months, and the median was 15.9 months. Rank-sum test showed that patients in the EP300_low_ group had a longer overall cumulative survival time than those in the EP300_high_ group (*p* = 0.048, **Figure 3D**). Kaplan- Meier survival analysis showed that in different gender, age, lymph node metastasis and smoking history groups, there were no differences in cumulative survival time between EP300_low_ and EP300_high_ patients. However, in the tumor grade 3, the patients with low EP300 levels had a statistically longer cumulative survival time than those with high EP300 levels (*p* = 0.046, **Figure 3D**). Patients without history of alcohol consumption with low EP300 levels had a longer cumulative survival time than those with high EP300 levels (*p* = 0.028, **Figure 3D**). The patients in pathological T3 group with low EP300 levels had a longer cumulative survival time than those with high EP300 levels (*p* = 0.048, **Figure 3D**).

### EP300 knockdown inhibited cell proliferation and colony formation, as well as cell migration and invasion in ESCC

**Figure 4:**
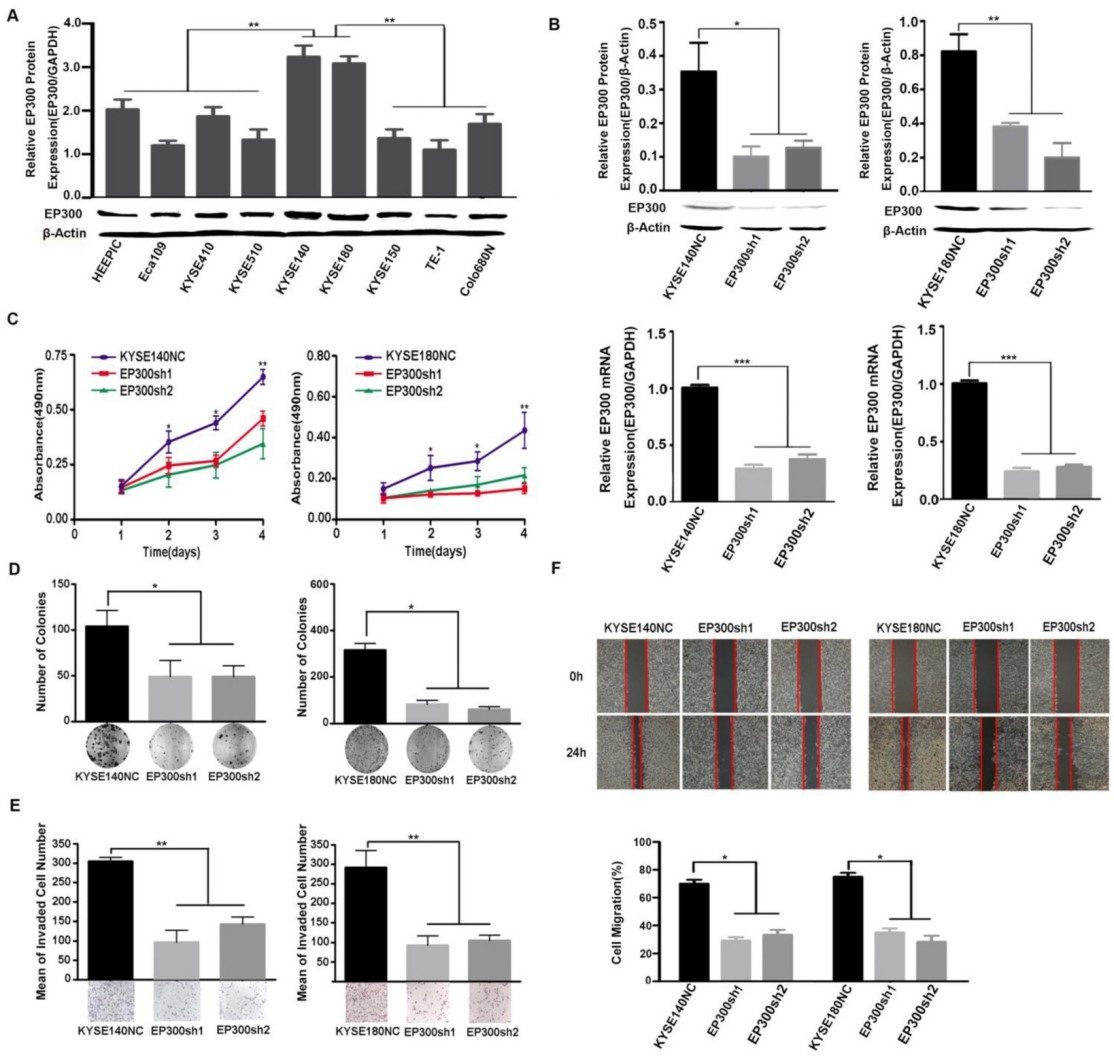
EP300 acts as an oncogene affecting ESCC cell growth, colony formation, cell migration and invasion. **(A)** The protein expression pattern of EP300 in nine ESCC cell lines detected by western blot. **(B)** Knockdown efficiency of EP300 in KYSE140 and KYSE180 cells were tested by western blot (up) and qPCR (low). **(C)** EP300 knockdown inhibited the proliferation of KYSE140 and KYSE180 cells by MTT assay. **(D)** The number of colony formation in ESCC cells was decreased in EP300 knockdown group compared to the control. **(E)** EP300 knockdown markedly inhibited KYSE140 and KYSE180 cell invasion. **(F)** Cell-migration monitored by wound healing assay was carried in KYSE140 and KYSE180 cells. The wound area was calculated using Image J software. All datas are presented as the mean ± standard deviation and three independent experiments. *p < 0.05, **p < 0.01, ***p < 0.001.

We measured EP300 protein levels in nine ESCC cell lines by western blot and found different basal expression levels in various ESCC cell lines. Of these cell lines, KYSE140 and KYSE180 cell lines with high endogenous EP300 level were used for knockdown experiments (**Figure 4A**). To investigate the function of EP300 in tumorigenesis, depletion of endogenous wild-type EP300 were carried out in KYSE140 and KYSE180 cell lines. The efficiency of knockdown was confirmed by qPCR and western blot respectively (**Figure 4B**). We observed that EP300 knockdown significantly inhibited proliferation of KYSE140 and KYSE180 cells as monitored by MTT and colony formation assay (*p* < 0.05,** Figure 4C- 4D**). Similarly, migration and invasion of KYSE140 and KYSE180 cells were also inhibited by EP300 knockdown as monitored by wound healing and transwell assay (*p* < 0.05, **Figure 4E-4F**). These results suggested that EP300 might possess tumor promoting effect in ESCC.

### Altered pathways and gene-interaction networks affected by EP300-knockdown in ESCC cells

**Figure 5:**
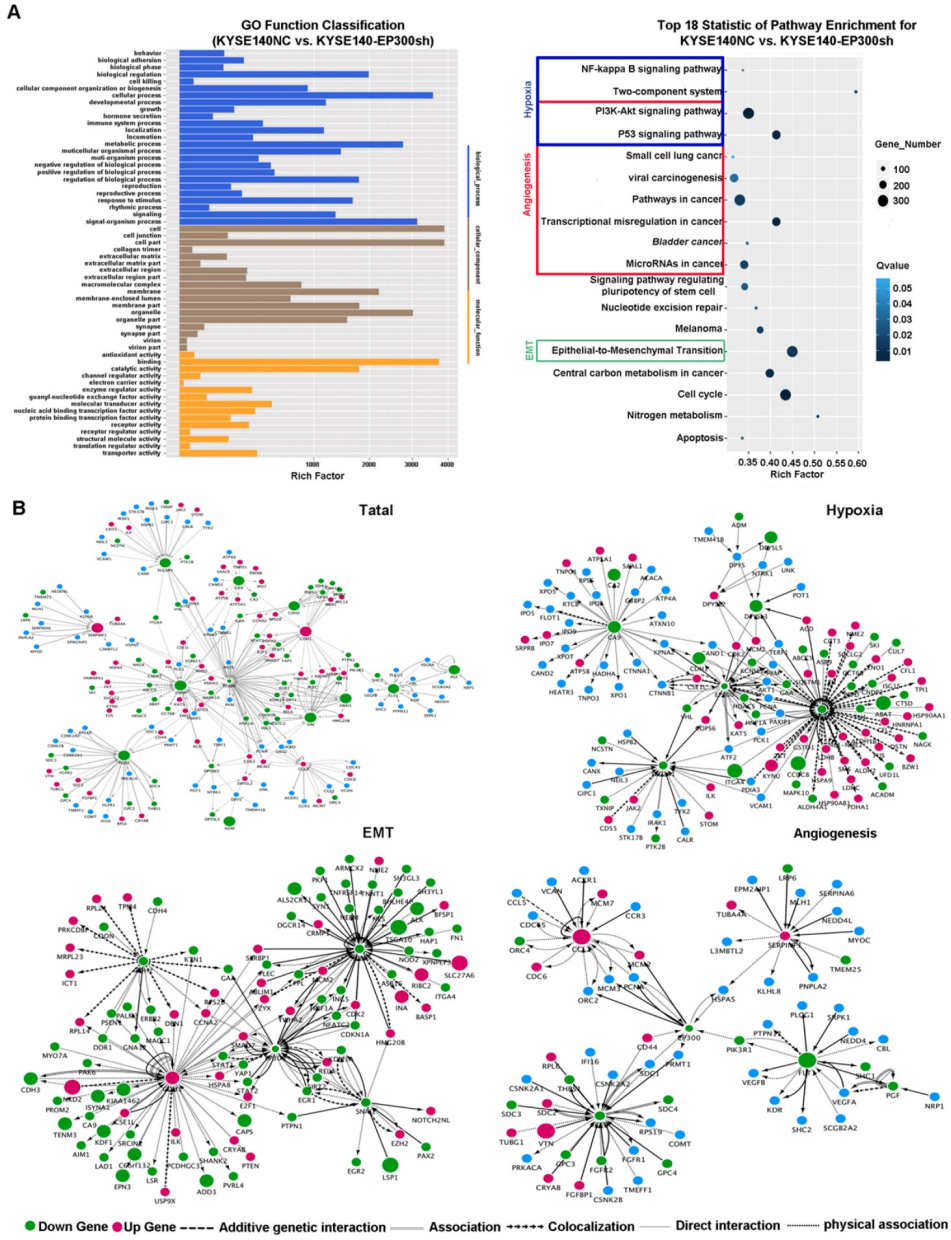
Key cancer pathway components altered in EP300 knockdown cells and gene-interaction networks of EP300. **(A)** Left is the GO analysis of annotation entries of significantly differentially expressed genes. Gene Ontology includes three ontologies, the biological process (blue), cellular component (brown), and molecular function (yellow). Right is the pathway-enrichment analysis of differentially expressed genes. **(B)** Network of EP300 interactors according to RNA-seq (up and left). Network of EP300 interactors focus on hypoxia pathway (up and right), EMT pathway (low and left) and angiogenesis pathway (low and right).Up-regulated genes were represented by red; down-regulated genes were represented by green; blue represented no significant difference. The circle size represented fold change.

In order to explore the biologic mechanism of EP300 in ESCC tumorigenesis, RNA-Sequencing method was performed in EP300 knockdown KYSE140 and control cells to distinguish the differentially expressed genes. Gene ontology analysis displayed that the differentially expressed genes with *p* value lower than 0.05 were enriched in biologic processes including biological adhesion, biological regulation, cellular process, development process, metabolic process and so on (**Figure 5A**). The pathway enrichment analysis showed that these differentially expressed genes were enriched in the signaling pathways including PI3K-Akt signaling pathway, NF-kappa B signaling pathway, two-component system, pathways in cancer, transcriptional dysregulation in cancer, microRNAs in cancer and EMT indicating that EP300 may contribute to ESCC tumorigenesis via these pathways (**Figure 5A**).

Furthermore, we used cytoscape software to depict the association between differentially expressed genes and EP300. As shown in **Figure 5**, the network of the gene relationship was established according to known and predicted protein-protein interaction, and genes were clustered by different biological mechanism, including angiogenesis, hypoxia and epithelial-to-mesenchymal transition (EMT) (**Figure 5B**). In angiogenesis network, except for the central EP300 gene, there are other core genes including up-regulated SERPINF1 and down- regulated FGF2, FLT1, CCL2 (**Figure 5B**). In hypoxia network, core genes included down-regulation of LDHA, ADM, SLC2A1 and CA9 (**Figure 5B**). In EMT network, core genes also covered the up-regulation of E-cadherin (ECAD), down-regulation of N-cadherin (NCAD), Snail and Vimentin (**Figure 5B**).

**Figure 6:**
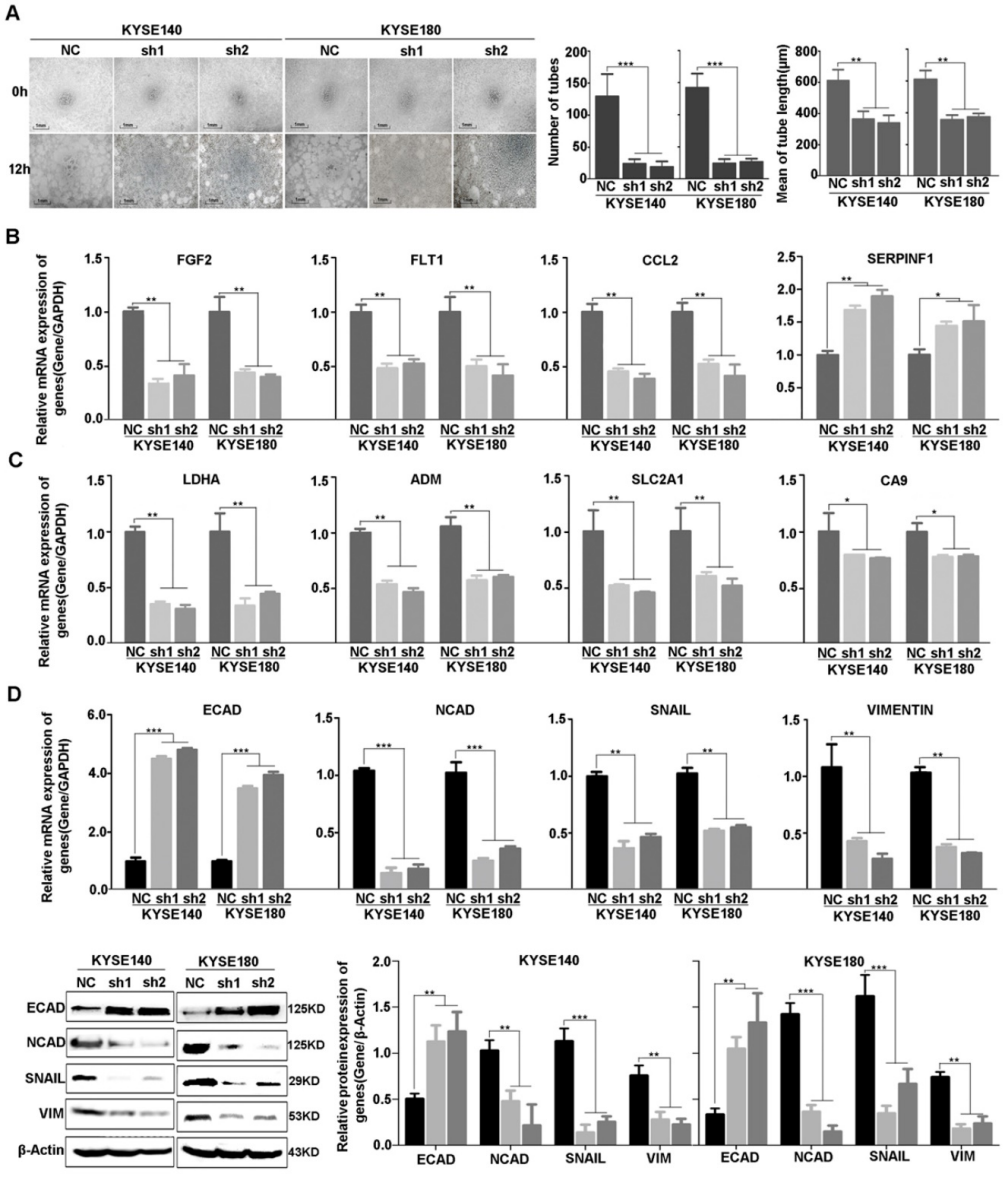
Effect of EP300 knockdown on angiogenesis of ESCC cells and the expression change of angiogenesis, hypoxia and EMT relevant molecules. **(A)** The left is the representative images of in vitro vasculogenic mimicry tube formation assay. The medium are the quantification results of in vitro vasculogenic mimicry tube formation using Image J software. The right are the quantification results of in vitro vasculogenic mimicry tube length using Image J software. **(B)** q-PCR was used to detect the mRNA level of FGF2, SERPINF1, FLT1 and CCL2 in KYSE140NC, KYSE180NC and EP300sh. **(C)** q-PCR was used to detect the mRNA level of LDHA, ADM, SLC2A1 and CA9 in KYSE140NC, KYSE180NC and EP300sh. **(D)** qPCR was used to detect the mRNA level of E-cadherin, N-cadherin, Snail and Vimentin in KYSE140NC, KYSE180NC and EP300sh. Western blot was used to detect the level of ECAD, NCAD, SNAIL and VIM in KYSE140NC, KYSE180NC and EP300sh. β-Actin was used as a loading control. All data are presented as the mean ± standard deviation and three independent experiments. *p < 0.05, **p < 0.01, ***p < 0.001.

Consistent with the result of RNA-sequencing and network analysis, we used vasculogenic mimicry in vitro to determine whether EP300 mediate the morphological alteration of the ESCC cells. Our results demonstrated that angiogenesis was inhibited after EP300 knockdown in ESCC cells, directly indicating the promotion effect of EP300 on angiogenesis (**Figure 6A**). Furthermore, we used quantitative real-time PCR (qPCR) and western blot methods to validate expression change of these genes at mRNA and protein levels. The results showed that a set of angiogenesis, hypoxia, and EMT related molecular markers were affected after knockdown of EP300 in ESCC. In details, the decrease of FGF2, FLT1, CCL2 mRNA, along with increase of SERPINF1 mRNA were observed in EP300 knockdown ESCC cells, predicting that EP300 may promote tumor initiation via angiogenesis in ESCC (**Figure 6B**). At the same time, LDHA, ADM, SLC2A1 and CA9 were also decreased in EP300 knockdown ESCC cells, indicating that EP300 may promote tumor growth and progress via hypoxia in ESCC (**Figure 6C**). Also, as shown in **Figure 6D**, the markedly up-regulation of E-cadherin and down-regulation of N-cadherin, Vimentin and Snail were observed in EP300 knockdown ESCC cells. Further western blot analysis also confirmed the expression change of these EMT related markers in EP300 knockdown ESCC cells, indicating that EP300 may promote tumor progress via EMT in ESCC (**Figure 6D**).

## Discussion

In this study, we identified a series of histone modifier genes which were frequently mutated in ESCC by combining our genomic sequencing data with previous datasets. And we found that both mutation of EP300 and high expression of EP300 predicted a shorter survival time. We demonstrated that the knockdown of the acetyltransferase activity of EP300 reduced cellular proliferation, migration and invasion. The tumor promoting role of EP300 in ESCC may be achieved by regulating the biological process including angiogenesis, hypoxia and EMT.

In our integrated analysis of genomic sequencing data in 325 ESCC, approximately 60% of ESCC tumors contained at least one chromatin remodeling gene alteration. Dysregulation of these encoded proteins possibly affected the gene expression at the genome-wide level and played key roles in DNA repair and genome maintenance 11. Obviously, inactivating and truncating mutations were observed in the MLL2 and MLL3 genes, members of MLL family and possessing histone methylation activity. It is well acknowledged that the patterns of mutations in known oncogenes and tumor suppressor genes are remarkably characteristic and nonrandom. According to the the “20/20 rule in Vogelstein's study 12, > 20% of the recorded mutations in oncogenes are usually at recurrent positions and are missenses. Whereas tumor suppressor genes are mutated through protein-truncating alterations throughout their length, meaning that > 20% of the mutations are inactivating. Thus, the mutation pattern of MLL2 and MLL3 may indicate the tumor-suppressor role in ESCC, which is supported by a large number of researches 13-15. In terms of EP300, 19% of the mutations were at the identical amino acid, codon 4195 and 32% the mutations were concentrated in three sites (codon 4195, 4241, 4540). Meanwhile, 19% of the mutations of EP300 were inactivating. So, it is difficult to judge the characteristic of EP300 in ESCC. Consistent with this is that there is evidence indicating that EP300 can function both as a tumor suppressor protein and as an oncoprotein 16, 17.

EP300 encodes the adenovirus E1A-associated cellular p300 transcriptional co-activator protein which functions as histone acetyltransferase that regulates transcription via chromatin remodeling and plays an important role in the processes of cell proliferation and differentiation. Here, our results in vitro in ESCC cell lines shows that EP300 function as an oncogene exerting tumor promotion effects, which has been demonstrated in lung, colon, prostate and breast cancers^18^-^21^. In contrast to our results, Gao' study showed that depletion of EP300 significantly increased tumor cell proliferation in two ESCC cell lines and ectopic expression of wild- type EP300 reversed this effect 6. This discrepancy is probably attributed to different genetic background of cell lines and further research is needed in ESCC. However, in regard to expression, we found it was overexpressed in ESCC tissues compared to that of matched adjacent tissues. And we also found that both mutation of EP300 and high expression of EP300 are associated with poor prognosis in ESCC, which was highly in accordance to Gao' results that EP300-mutated and overexpressed tumors had a dismal overall survival and Li's results showing that high expression EP300 was correlated with aggressive features and poor prognosis 6, 22. In addition, EP300 expression was found to be predictive for poor prognosis of prostate cancer, colon cancer and hepatocellular carcinoma patients and be correlated with tumor recurrence in breast cancer 18-20, 23. Interestingly, some researchers observed EP300 cytoplasmic location in cancer cells such as breast cancer and osteosarcoma cells 24, 25, but in our study here, EP300 protein showed strong nuclear staining in ESCC. It has been shown that EP300 distribution between the nucleus and cytoplasm may be modulated and that its cytoplasmic localization may be associated with a specific biological activity such as playing a role in p53 degradation 26. The nuclear staining of EP300 in our study may indicated the role of histone modification whereby regulating the expression of downstream molecules and participating in the associated biologic process.

Accordingly, our RNA-sequencing results and validation tests indicated that EP300 may contribute to ESCC tumorigenesis via angiogenesis, hypoxia and EMT signaling pathways. We deduce that EP300 interacts with sequence-specific transcription factors and promotes activation of angiogenesis, hypoxia, EMT relevant genes, whereby participating in the tumorigenesis and development of ESCC. The changed genes in angiogenesis in EP300 knockdown ESCC cells included the down- regulation of FGF2, FLT1, CCL2, and up-regulation of SERPINF1. It has been reported that SERPINF1 protein strongly inhibits angiogenesis 25. FLT1 can bind to VEGFR-A, VEGFR-B and FGF2 and play an important role in promoting endothelial cell proliferation, survival and angiogenesis in Hepatocellular Carcinoma, Non-Small Cell Lung Cancer and Esophageal Cancer 26-29. These findings were consistent with the trend of expression change of these genes in our study indicating the promotion effect of EP300 on angiogenesis. Meanwhile, the varied genes in hypoxia included down-regulation of LDHA, ADM, SLC2A1 and CA9. Among them, LDHA is a HIF1α-targeted glycolytic genes and is reported to be up-regulated in various cancer cells 27, 28. ADM, potentially induced by hypoxia and also harboring promotion of angiogenesis, is highly associated with the prognosis and disease severity 29. CA9 is a target gene of HIF1α and functions as a part of the cellular response to hypoxia to regulate intracellular pH, thereby promoting cell survival 30. In our study, we also identified the expression change of EMT related markers including known E-cadherin, N-cadherin, Snail and Vimentin in EP300 knockdown ESCC cells.

It is well known that angiogenesis, hypoxia- inducible factor (HIF) reaction and EMT are three major biological processes that participate in the tumorigenesis. Hypoxia has been shown to cause metabolic and molecular changes in endothelial cells, and induces the imbalance between pro- and anti-angiogenic factors' production, leading to enhanced, rapid and turbid blood vessel formation. Moreover, HIF-α contributes to cancer metastasis by altering adhesion and motility ability of cancer cell through regulation of epithelial-mesenchymal transition 31, 32. Our results possibly indicated that EP300 might be a key central regulator in angiogenesis, hypoxia and EMT pathway, forming a complex regulatory network and playing an important role in ESCC 33, 34.

In summary, we used genomics data to show the link between histone modifier gene mutations and ESCC. Our functional and clinical analyses show that deletion of EP300 inhibits angiogenesis, hypoxia and EMT process, and high expression and mutation of EP300 correlates with poor prognosis, thus it may act as an oncogene in ESCC. Our findings have important implications for understanding the mechanisms that drive the development and progression of ESCC and may provide potential therapeutic targets for ESCC treatment.

## Materials and Methods

### Samples and clinical data

Tumor and adjacent normal tissue samples of patients were obtained from 104 ESCC affected individuals recruited from the ethics committee of Shanxi Cancer Hospital and Henan Cancer Hospital. The ESCC individuals collected for this study were staged according to the Cancer Staging Standards of the American Joint Committee on Cancer (seventh edition, 2010). 104 individuals were assayed on each platform: 14 tumors and matched normal samples experienced WGS and 90 samples experienced WES. Sequencing data and clinical characteristics of the analyzed samples were presented in our previously published study and available for download from the European Genome-phenome Archive (EGA) under accession number EGAS00001001487 3. Another three cohorts were from Song's report of 17 WGS and 71 WES ESCC samples recruited from the Chaoshan District, Lin's study of 20 WES ESCC patients from Cancer Institute/Hospital, Chinese Academy of Medical Sciences (CAMS) and Linxian Cancer Hospital, and Gao's report which contained 113 WES samples recruited from CAMS 4-6.

### Cell lines

All esophageal cancer cell lines including KYSE140, KYSE180, ECA109, KYSE410, KYSE510, KYSE150, TE1 were preserved in Translational Medicine Research Center of Shanxi Medical University (Taiyuan, China). All cells were grown in RPMI 1640 medium (Hyclone, Logan, UT, USA) supplemented with 10% fetal bovine serum (FBS, Gibco, Thermo Fisher Scientific) at 37 °C in 5% CO2.

### Real-time quantitative PCR (qPCR)

qPCR was used for measuring expression levels of genes of interest in ESCC cell lines. Total RNA was extracted from cells using the RNA extraction reagent (RNAiso Plus, Takara, Bio Inc, Japan). qPCR was performed using the SYBR Green Premix Ex TaqTM (catalog no. RR820A, Takara Bio Inc, Japan) following the manufacturer's protocol. All qPCR reactions were performed in triplicate with an Applied Biosystems StepOnePlus. The relative expression of genes of interest was determined by normalization to GAPDH expression according to the manufacturer's instructions. All real-time PCR experiments included a no-template control and were done in triplicate.

### Knockdown of EP300 in ESCC cell lines

Knockdown EP300 was performed in KYSE140 and KYSE180 cell lines with high endogenous protein levels. Two independent shRNAs were cloned into the pLKO.1-puro vector. We transfected HEK293T cells with the packaging plasmids pMD2.G and psPAX2 along with the lentiviral shRNA vector by using Lipofectamine 2000 reagent (Invitrogen) to produce virus production. We harvested virus 48hr after transfection, passed through 0.22-μm filters, and performed lentiviral infections with the appropriately tittered fresh viral supernatant. The target ESCC cells were infected at 50%-60% confluence and incubated at 37°C for twenty-four hours, and the viral supernatant was replaced with fresh media. Infected cells were screened in 4 mg/ml puromycin 48 h later. After one week, the efficiency of shRNA knockdown was detected by western blot and qPCR analysis.

### MTT assay

Cultured cells were digested for the preparation of single-cell suspensions, which were seeded at a density of 5 × 10^3^/well into 96-well plates, and incubated overnight in normal conditions. After 24hr, 48hr, 72hr and 96hr, cells were treated with 20μl of 5 mg/ml of MTT (Invitrogen) solution and incubated 4 h at 37°C. Before addition of 150μl of DMSO to each well to dissolve the crystals, the MTT solution was discarded. The absorbance was measured with an ELISA reader at 490 nm. Each experiment consisted of five replications, and repeated at least three times.

### Migration and invasion assays

Transwell migration and invasion assays were performed to evaluate the effect on cell migration and invasion. 5 × 10^4^ cells were plated into the upper chamber of a 24-well plate and cultured with FBS freed medium. The lower chambers were filled with 600μl RPMI 1640 containing 10% FBS. The plate was incubated for 48hr, the cells were fixed with 4% paraformaldehyde and stained with 0.1% crystal violet. Microscopy (Olympus, Japan) was used to image the cells that transmigrated to the underneath surface of the transwell membrane. Randomly selected five fields of transmigrated cell versions and counted manually. For the transwell invasion assays, the membrane was precoated with 50μl of Matrigel (1:6 mixed with FBS freed RPMI 1640; BD Biosciences, Heidelberg, Germany) and proceeded the same as described above.

### Wound healing assay

A total of 2 × 10^5^ cells were seeded into 6-well dishes, and when they reached 90% confluence, a scratch was created with a 200μl pipette tip. Subsequently, cells were cultured in a serum freed RPMI 1640 medium at 37°C in 5% CO2 for the next 48hr. Micrographs were captured at 0hr and 48hr. Three separate studies were conducted.

### Colony formation assay

Cells were seeded at 500 cells per well in 6-well plates containing complete RIPM-1640 and allowed to grow for 2 weeks. On day 14, cells were fixed with 4% paraformaldehyde and stained with 1% crystal violet before manually counted.

### Production of tissue micro-array (TMA) and immunohistochemistry analysis

Tissue chip production as follows, we prepared blank receptor wax block and extract tissues from donor wax block. Next, we put the donor tissues into corresponding holes of the blank receptor wax block and repeated freezing and thawing the new wax block to make them together. Immunohistochemistry (IHC) was performed for the detection of EP300, sections were incubated with special antibody (Abcam) at an ideal dilution for overnight at 4°C. Then we used PV8000 (Zhongshan) and the DAB detection kit (Maixin) to detect the interested protein of slides, and hematoxylin was used to counterstain them. All images were captured at 1003. The amount of the protein of interest was analyzed with Aperio Nuclear v.9 software. Statistical analyses were performed with SPSS 19.0.

### RNA-sequencing

Total RNA was extracted from cell lines using the TRIzol reagent (Life Technologies, Carlsbad, CA, USA) and DNA was digested by DNase I following the instructions of manufacturer. RNA quantity and quality was evaluated by NanoDrop spectrophotometer (Thermo Scientific, USA). 1% gel electrophoresis was used to determine the RNA integrity. Enriched mRNA with Oligo (dT), and broke them into fragments for the preparation of cDNA libraries. The cDNA libraries were quality inspection qualified with the Agilent 2100 Bioanalyzer and ABI Step One Plus Real-Time PCR System, then sequenced on Illumina HiSeqTM 2000 at BGI (The Beijing Genomics Institute), Shenzhen, China.

### Western blot

Western blot analysis experiments were conducted at least three times. Total protein was extracted using RIPA buffer (Sigma) containing protease and phosphatase inhibitors (Thermo Fisher Scientific) on ice for 1hr. 50 μg of protein was separated using SDS-PAGE (10% separating gel and 5% stacking gel) and then transferred to nitrocellulose filter membranes (Whatman GmbH, Maidstone, Kent, UK). The membrane was incubated blocked with 5% non-fat milk for 2 hr at room temperature, then incubated with special antibodies at 4°C overnight. The blot was detected with horseradish peroxidase labeled secondary antibody (Sigma), and chemiluminescence was detected with a LAS4000 device (Fuji). β-Actin (Proteintech, Group, Wuhan, China) was used as loading control.

### Vasculogenic mimicry

50μl matrigel matrix was seeded in the wells of a 96-well plate and incubated at 37°C for 30min to promote solidification. 5 × 10^5^ cells KYSE140NC, KYSE180NC, KYSE140-EP300sh and KYSE180sh cells were seeded into the gel in 50μl complete DMEM medium (Lonza, Walkersville, USA) incubated at 37°C for 12hr. We selected 10 views randomly, the formation of tube-like structures was captured under an inverted light microscope, and the tube number and tube length were measured with Image J software.

### Bioinformatics analysis

Primary sequencing data that produced by Illumina HiSeqTM 2000, called as raw reads, is subjected to quality control (QC) that determine if a re-sequencing step is needed. After QC, raw reads are filtered into clean reads which will be aligned to the reference sequences. QC of alignment is performed to determine if re-sequencing is needed. The alignment data is utilized to calculate distribution of reads on reference genes and mapping ratio. If alignment result passes QC, we will proceed with downstream analysis including gene and isoform expression, deep analysis based on gene expression (PCA/ correlation/screening differently expressed genes and so on), exon expression, gene structure refinement, alternative splicing, novel transcript prediction and annotation, SNP detection, indel detection. Further, we also can perform deep analysis based on DEGs, including Gene Ontology (GO) enrichment analysis, Pathway enrichment analysis, cluster analysis, protein-protein interaction network analysis and finding transcription factor. All the pictures were plotted by cytoscape.

### Statistical analysis

All participants and various subgroup patients were separated into EP300WT and EP300mutant groups, or EP300_high_ and EP300_low_ based on ROC curves. Rank sum and Chi square (χ2) tests were carried out to compare differences of EP300 expression levels in ESCC tissues with various clinic-pathological parameters. Kaplan-Meier estimation and Log-rank test were used to compare cumulative survival time between different EP300 groups with clinic-pathological factors. Univariate and multivariate survival analysis were performed by a Cox proportional hazards regression model. All datas were processed using the SPSS 19.0 statistical software program (IBS SPSS, Armonk, NY, USA). A difference with the p value less than 0.05 was considered statistically significant.

## Supplementary Material

Supplementary table.

## Figures and Tables

**Table 1:** Correlation analysis between EP300 protein levels in ESCC and clinicopathological variables

Characteristics	Total (n=65)	Rank sum tests		EP300 expression [n(%)]		Chi-square test	
		Z/χ2	P-value	Low (T<122.388) n=45	High (T≥122.388) n=20	χ2	P-value
Age							
<60	37	-0.338	0.735	26(70.270)	11(29.730)	0.044	0.835
≥60	28			19(67.857)	9(32.143)		
Gender							
Male	45	-1.514	0.130	27(60.000)	18(40.000)	5.850	0.016*
Female	20			18(90.000)	2(10.000)		
smoking							
Yes	42	-1.598	0.110	25(59.524)	17(40.476)	5.250	0.022*
No	23			20(87.000)	3(13.000)		
Drinking							
Yes	23	-0.727	0.467	14(60.870)	9(39.130)	1.168	0.280
No	42			31(73.810)	11(26.190)		
Family history							
Yes	16	-0.265	0.791	12(75.000)	4(25.000)	0.070	0.792
No	49			33(67.347)	16(32.653)		
lymphatic metastasis							
No	15	-1.495	0.135	13(86.667)	2(13.333)	1.821	0.177
Yes	50			32(64.000)	18(36.000)		
Tumor grade							
2	44	-0.231	0.817	30(68.182)	14(31.818)	0.070	0.791
3	21			15(71.429)	6(28.571)		
Pathological T							
T1+T2	11	-1.942	0.052	9(81.818)	2(18.182)	0.556	0.456
T3	54			35(64.815)	19(35.185)		
TNM Stage							
Ⅰ+Ⅱ	17	-0.925	0.355	14(82.353)	3(17.647)	1.861	0.173
Ⅲ	48			31(64.583)	17(35.417)		

*p < 0.05
