# Supplementary material for: EP300 as an oncogene correlates with poor prognosis in esophageal squamous carcinoma
Source: J Cancer. 2019 Aug 29;10(22):5413–26. doi: 10.7150/jca.34261 (PMC6775682; doi:10.7150/jca.34261)
Supplement: Supplementary file 1 — Supplementary table. [file jcav10p5413s1.pdf]

Supplementary Table 1: Univariate and multivariate Cox models for the correlation between survival and clinicopathological factors in patients with ESCC

Variables		Univariate analysis			Multivariate analysis		
		HR	95% CI	P-value	HR	95% CI	P-value
Gender	Female vs.Male	0.987	0.692-1.407	0.942	1.056	0.699-1.596	0.796
Age	＞60 vs. ≤60	0.967	0.752-1.242	0.790	0.962	0.746-1.241	0.767
Smoking status	yes vs. no	1.110	0.846-1.456	0.453	1.254	0.911-1.726	0.165
Lymph node metastasis	yes vs. no	2.148	1.636-2.820	**0.000**	2.027	1.537-2.673	**0.000**
Tumor grade	2 vs.1	1.326	0.974-1.804	0.073	1.207	0.882-1.650	0.240
	3 vs.1	1.798	1.234-2.6221	**0.002**	1.564	1.067-2.294	**0.022**
Pathological T	2 vs.1	1.378	0.698-2.720	0.355	1.234	0.621-2.455	0.548
	3 vs.1	1.696	0.921-3.122	0.090	1.423	0.768-2.640	0.263
	4 vs.1	3.642	1.829-7.252	**0.000**	3.177	1.583-6.377	**0.001**
EP300	mutant vs.WT	1.668	1.062-2.621	**0.026**	1.809	1.143-2.864	**0.011**
